# “Attending lectures in your pyjamas”: student agency in constrained circumstances

**DOI:** 10.1007/s10734-022-00976-9

**Published:** 2022-12-15

**Authors:** Rola Ajjawi, Juan Fischer, Joanna Tai, Margaret Bearman, Trina Jorre de St Jorre

**Affiliations:** 1 Centre for Research in Assessment and Digital Learning (CRADLE), Deakin University, Melbourne, VIC Australia; 2 Student Experience, University of New England, Armidale, NSW Australia

**Keywords:** Agency, Undergraduate, Emergency remote teaching, Study

## Abstract

COVID-19 forced the digitalisation of teaching and learning in a response often described as emergency remote teaching (ERT). This rapid response changed the social, spatial, and temporal arrangements of higher education and required important adaptations from educators and students alike. However, while the literature has examined the constraints students faced (e.g. availability of the internet) and the consequences of the pandemic (e.g. student mental health), students’ active management of these constraints for learning remains underexplored. This paper aims to “think with” COVID-19 to explore student agency in home learning under constrained circumstances. This qualitative study used semi-structured interviews to understand the day-to-day actions of nineteen undergraduate students managing their learning during the COVID-19 lockdowns in Victoria, Australia. Emirbayer and Mische’s multiple dimensions of agency — iterative, projective, and practical-evaluative — are used to explore student experience. The findings illustrate students’ adaptability and agency in navigating life-integrated learning, with most of their actions oriented to their present circumstances. This practical evaluative form of agency was expressed through (1) organising self, space, time, and relationships; (2) self-care; and (3) seeking help. Although this study took place in the context of ERT, it has implications beyond the pandemic because higher education *always* operates under constraints, and in other circumstances, many students still experience emotionally and materially difficult times.

## Introduction

Cultivating student agency is a key part of transitions to new learning environments (Charteris & Smardon, 2017), and examining student responses to changing learning and teaching environments during the COVID-19 pandemic may shed light on this process. Agency is about purposeful action; students can act, construct, or contribute in some form with respect to their studies. When COVID-19 forced higher education to switch to emergency remote teaching (ERT), teachers and students adjusted their practices in a relatively short timeframe. While the literature has examined the impact of the pandemic in many different spheres, how students actively managed their new learning environment remains underexplored. This is not just a retrospective view: studying student agency during COVID-19 may offer significant insights into how students can negotiate their learning more generally.

The premise of this paper is to “think with” COVID-19 about student agency in home learning. We follow Jensen (2021, p. 66) who suggests that “the Covid-19 event is as much a window into the existing and ordinary practices, as into the extraordinary and future ones”. Thus, we seek to “think with” COVID-19 by considering what insights can be drawn from a study context set in ERT, aiming to understand how students wrangle studying within unusually constrained space and time. This has implications beyond the pandemic because higher education *always* operates under constraints. From an institutional perspective, there are limited resources to invest in supporting an increasingly inclusive approach to education. From the student perspective, an increasingly diverse student body manages a broad range of lifeworld commitments, such as caring responsibilities, or juggling work, children, and home. Thus, students must negotiate learning from a range of different and unequal positions (Bennett & Burke, 2017), and they do so in circumstances where the time and space for study can be very limited. By investigating what students *do* within material and temporal challenges set by the pandemic, we can better understand how diverse students manage the difficulties of limited social and material conditions, and how institutions can offer more appropriate support across a broad student cohort.

### Research in emergency remote teaching

ERT research with students has primarily focused on their affective responses such as anxiety/fear (Cao et al., 2020; Chen & Lucock, 2022), lower motivation (Tasso et al., 2021), and lower overall satisfaction (Means & Neisler, 2021) at least early during ERT (Loton et al., 2020). It has also identified the varied contextual constraints to learning thrown up by ERT. The shift to studying at home necessitated different arrangements, as spaces “… carry particular values and histories, which in turn influence how people act within them” (Variyan & Reimer, 2021, p. 6). Study during ERT benefited from access to computers and fast internet connections (Castelli & Sarvary, 2021; Rizvi & Nabi, 2021; Weldon et al., 2021), as well as to adequate study spaces and support from other people (McKay et al., 2021; Rizvi & Nabi, 2021; Wilson et al., 2020). Students from lower socioeconomic backgrounds were more affected as they lived in areas with poorer internet coverage or connectivity (Cullinan et al., 2021), a reason cited for why students do not turn on their cameras during video tutorials (Castelli & Sarvary, 2021). These studies contribute to framing student experience beyond their individual perceptions and study approaches, by anchoring them to their local social and material conditions. Therefore, research has so far identified ERT environmental constraints and its affective consequences on students, but not how these students wrangled their study as part of their response to these conditions.

Several ERT studies explored student agency, although none directly addressed how students negotiated studying. Camfield et al. (2021) employed the lens of student self-efficacy and noted that while students lost some sense of agency and belonging, they recovered as time went on with inputs from their instructors. Similarly, McKay et al. (2021) showed that first-year student wellbeing and engagement initially dropped with ERT and lockdown but recovered through personal strategies like humour, making strategic decisions, and actively seeking help from others. In addition, Wilson et al. (2020), through an autoethnographic approach, showed how self-awareness and self-accountability were important to reduce students’ feelings of disconnection, while highlighting that such self-regulation is mediated by emotions and external factors. These include the structural influences of the university, as well as the presence/absence of peers, and the home environment where distractions abound, while social interactions and structuring cues of everyday life are scarce. These studies highlight students’ feelings of agency, and how that agency is enabled or constrained by the environment, but it remains unclear how students agentically mobilised their resources to support their studies.

While teacher agency in times of COVID has been studied and shown to be variable (Damşa et al., 2021; Variyan & Reimer, 2021), student agency within the constraints of ERT has not. Student experiences within ERT highlight digital inequity such as access to working computers, strong internet connection, and productive spaces from which to study (Bashir et al., 2021; Guppy et al., 2022). We suggest that challenges will continue to affect students, particularly as technologies and hybrid learning further penetrate higher education. Thus, understanding how students negotiate the challenges of home learning environments is important to building better education systems.

### Theoretical framing of agency

While there are multiple conceptions of agency in higher education research, we follow Emirbayer and Mische’s (1998) definition because of the interplay of agency with structural and temporal dimensions already noted. Agency can be considered as follows: “the temporally constructed engagement by actors of different structural environments … which, through the interplay of habit, imagination, and judgment, both reproduces and transforms those structures in interactive response to the problems posed by changing historical situations” (p.970). Agency is bounded; one acts within certain constraints imposed by previous experience as well as context. Emirbayer and Mische (1998) propose a comprehensive relational framework, acknowledging multiple dimensions of agency. The three constitutive analytic dimensions are iteration (habit), projectivity (imagination), and practical evaluation (judgement). Iterative agency is the routinised and habitual activity (thought and action) shaped by prior experiences. Projective agency is imaginative involving reconfiguring of thoughts and actions to future trajectories, hopes, and desires. As the name suggests, practical-evaluative agency is the practical judgment about alternative trajectories in the face of dilemmas and change.

The temporal plays out in each of these forms of agency as the past, future, and present, with all three analytical dimensions always present but not necessarily in balance or harmony. “The ways in which people understand their own relationship to the past, future, and present *make a difference* to their actions” (Emirbayer & Mische, 1998, p. 973); an individual’s agentic orientation is mutable. Intuitively, we can see the relationship between this view of agency and the constraints manifested during ERT, which included reconfiguring of physical learning spaces (the material nature of things), activity spatialities (a place to perform the activity), and places of connection (a sense of place) fundamentally changing the ecologies of higher education (Schatzki, 2021). These changes to study practices suggest that past temporal and physical routines of learning were no longer suitable during ERT requiring adaptation. While previously some students may not have chosen to study online, this was no longer a choice with ERT and lockdowns. Times of change, therefore, offer an opportunity to examine how students responded to changing time, space, and routine. How did students adapt their routines or project alternative futures for a problematic present or practically improvise around the demands of the present?

## Methods

This study of student agency is framed within a qualitative investigation of undergraduate students’ actions in managing remote learning during the 2020 COVID-19 lockdowns in Victoria, Australia. The focus was on their day-to-day actions supporting learning, not on measuring learning. Research ethics was granted by the Human Ethics Advisory Group of the Faculty of Arts & Education at Deakin University. As the COVID-19 experience could have been traumatic for some students, those who showed signs of concern, distress, or requiring further support were informed about the university’s confidential counselling and psychological support service, and about external organisations. Recruitment to this study occurred through a voluntary survey for a larger international project (Bartolic et al., 2022; Guppy et al., 2022). At the end of the survey, students could indicate their interest in taking part in an interview examining their experiences and actions during ERT. The interview data constituted a separate study to the large collaborations from which the students were recruited.

### Participants

Of 53 students who indicated willingness to partake in an interview, 19 undergraduate students of all year levels from one university were interviewed by RA, JT, and PM (see “Acknowledgements”) between 16 July and 12 August 2020, after they had completed one trimester of remote learning during the lockdown. Students were selected to maximise diversity in relation to the following characteristics. Fourteen participants identified as female and 5 as male, and their ages ranged 18–46 years old, with 15 students under 25. The students were undertaking diverse courses that included the following: business/commerce, science/health, civil engineering, arts/law/internal relations, and psychology, most of them enrolled on a full-time basis (14 of 19) and 15 were studying on campus before the pandemic, while 4 were already enrolled in remote courses before all courses transitioned to the remote format. Three of the 19 students were international.

### Data collection

The students participated in semi-structured interviews that ranged between 28 min and 1 h and covered topics about the transition to remote learning, and the day-to-day actions associated with studying and negotiating state-wide lockdowns. We aimed to establish the particular “structuring contexts of action” (Emirbayer & Mische, 1998, p. 1005) by eliciting narratives or stories of student resources and concrete descriptors of the environment including spaces, people, materials, and life. For example, “Can you tell me what a ‘regular’ day might look like? (Prompts may include: Where do you study, when, what room, who with, what with etc.)” and “have you established any routines to help you complete your studies? (Ask to elaborate)”. We asked for examples of people they connected with, actions that supported their wellbeing, and how they managed their emotions. We also asked how they imagined managing their home learning environments in the future and how the ERT experience has informed their understanding of themselves as learners moving forward. The wording of interview guide questions was refined after the first 2–3 interviews were conducted. Interviews were transcribed and all identifiable information was removed before the data analysis stage.

### Data analysis

Analysis of the interviews was an iterative process that started with all authors reading sample interview transcripts to become familiar with their content. We had intended to first identify the three dimensions of agency proposed by Emirbayer and Mische (1998) — *iterative*, *projective,* and *practical-evaluative *agency — through initial deductive coding and then to elaborate the interpretations of the data through inductive coding. However, data was too limited in the iterative and projective dimensions for further inductive coding but it was included in the overall data interpretation. Therefore, we proceeded with inductive coding only for the data coded as practical-evaluative forms of agency. Coding was at the level of sentences or stories to retain the contextual aspects that shape the possibilities of students’ agency, emphasising that it cannot be reduced to solely individual action. This involved in-depth reading of the data, discussions regarding patterns of activity across the data, and identification of sub-codes of the practical-evaluative agency. After designing the coding framework, each author coded one interview to test its usefulness and reflect on their interpretations, and one of the authors (JF) coded the remaining transcripts within the qualitative analysis software *NVivo* (2020), by QSR International. The team met several times to discuss the coded data for all three forms of agency and developed preliminary interpretations which were further tested against the data as team meetings progressed and through writing.

## Findings

Emirbayer and Mische (1998) anticipated that all three of their constitutive dimensions of agency should be “found, in varying degrees, within any concrete empirical instance of action” (p. 971). They also proposed that “in any given case, one or another of these three aspects might well predominate” (p. 972). Our findings are consistent with both insights. Based on our interviews, student agency focused predominately on practical-evaluative agency, oriented mainly to the here-and-now. ERT surfaced more agency motivated by emerging demands that required immediate, hands-on responses. Student actions were less oriented to iterative agency (habits and routines) and projective agency (possible futures), although both of these dimensions were visible in student responses. While we acknowledge the dynamic interplay between these dimensions, for the purposes of clarity, we describe each separately first, beginning with projective agency.

### Projective agency

*Projective* agency was the most limited dimension of agency identified in student data. Students hoped and aspired for a better future, one in which their careers would be better aligned to their dreams. This motivated current action. For example, students expressed hopes which drove action now: “when I graduate, I'll be able to get a job, and then that will benefit my family a whole heap” (S32). Similarly, S41 invoked wanting a career change as a strong motivator for action and persistence. These hopes were cited as motivation to study in the present. S29 projected that learning how to work and study from home would be valuable in the future. While some wanted to pause their study to avoid the online units, their future aspirations drove them to continue with their studies.

I had done an online unit before. That's why I know I'm not the best at online. [chuckles] I just did one unit online, my previous degree, and I found myself getting quite lost. I was a little bit “I don't know if this is going to be a good idea but what's the alternative?” The alternative would be withdrawing and then I wouldn't be able to follow the path that I want to go down. Not really an option. (S32)

Action in the present was spurred with hopes “to do well” (S6). Unsurprisingly there was “fear associated with the unknown” (S53), “an added layer of stress” (S6) and fear that practicals going online would “reduce the value of the degree” (S9). Some were anxious that the lack of face-to-face study would diminish their chance of being able to ask for a reference from the teachers. Emotional language imbued their future projections.

### Iterative agency

*Iterative* agency was also only lightly reported. ERT disrupted previous routines, necessitating students to establish new ones — which was not as straightforward as expected. S26 “struggled” to establish one and S36 reported:

I had a routine set up for my on-campus classes. When it transitioned to online, I had to change the routine completely, while also maintaining university and all the things that I had already committed myself to. I found it really difficult. I think my judgment of my capabilities was wrong because I thought that I would be able to cope quite well with it, but when it actually came to it, I didn't cope as well as I thought. (S36)

Students described having particular routines that remained the same — replication — for accessing discussion boards or group assignments or online quizzes, taking notes, and sending emails, particularly for those who had studied online before.

I had known how to research all of my assignments and articles beforehand, and in the transition to online I didn't see any guides or anything on how to do that, and so a lot of people were struggling. But I could just take the skills that I had already learned and apply them. (S24)

Others drew from their established work routines (outside of university) around online meetings and utilised these in their study: “You get comfortable with sitting in meetings just with heads or even no faces” (S7). S29 notes: “The knowing of how and where everything is. In terms of assessments, it did get easier”.

Being in first year posed more problems with iterative agency. For example, continuing students spoke of existing social connection:

I suppose it’s easier if you’d gotten to know some people, opposed to if you’d been in first year where you hadn’t got to know anyone yet. That was certainly made a bit easier, being able to at least contact them and see if they had any idea if you were confused on something as well (S47).

Others spoke of pre-established routines: “I've been to uni before, so it's not like I didn't know where to find resources and stuff” (S3).

### Practical-evaluative agency

Students’ *practical-evaluative* agency was extensively described and exercised by participants with patterns of actions within, and around, the new constraints imposed by COVID-19 and ERT. We interpreted three intersecting and overlapping categories: (1) organising self, space, time, relationships; (2) self-care; and (3) seeking help.

#### Organising self, space, time, and relationships

One of the challenges of ERT was that students who had previously studied on campus were missing the social and spatial cues that organised their daily routines. S24 explained how it was “hard to get in the mood to study when you can just attend your lectures in your pyjamas” melding together study and relaxing time, when in the past they saw the library and the campus as “a much better studying environment”. S32 seemed to enjoy the flexibility of studying on their own terms, but also reflected that while “[one] can just chill out”, it may not be the best way to study. Under these circumstances, most students found new ways to organise themselves, their time, and their personal space for responding to their study and work obligations. This included establishing new routines for studying at home such as setting a time for waking up and getting ready before dedicating given amounts of time to each subject and assignment. This structuring also allowed time for breaks and exercise. Moreover, many students created physical schedules to help them stay on top of tasks and deadlines, which could take the form of spreadsheets (S7), notes and timetables pinned to a wall (S9), or the printable Deakin planner (S32) which students accessed online. This manipulation of the physical space worked as a constant reminder for organising their own time, and on occasions, as a reminder to other people in the household that the student required study time and/or space.

This organisational work also involved setting up study spaces, which for some involved more challenges than for others. S47, for example, already had a desk since their school days and an additional screen to extend the laptop’s screen, while S32 required buying a new computer that could facilitate video conferences and ensure a stable internet connection, as well as changing the kitchen chair for a gym ball to be more comfortable during long classes. S41 was able to organise a studio in a spare bedroom, but it was one without natural light and reduced airflow — “I have all the doors open and try to get airflow because otherwise, it gets quite stuffy”. In contrast, S43 alternated between studying at the kitchen table or on their bed, “depending on the noise levels”, showing how the study space was rather improvised action in the immediate present to be renegotiated regularly.

Students negotiated with other people at home to create their study spaces, which did not always result in optimal conditions for study. S47, for example, intended to use the family studio for remote learning but ended up using the bedroom when their mother started working from home. This same student had moved back to the family’s farm during the lockdown and negotiated with their father that they needed to focus on studying and could only help in farm work during breaks. Meanwhile, S43 had to share their laptop with a sibling who was also studying at the same year level, which ended up in a less-than-ideal system: “We made a program, I guess, or whoever got to it first or whoever woke up first got to use it”. S32 used the physical timetable and a headset to signal “Mum’s study times” so her children would know when they should not interrupt, while S24 had to resort to wearing earplugs or listening to loud music as their two siblings would play guitar loudly, even during lectures.

#### Self-care

Students also wrangled themselves, their space, and their time for self-care and wellbeing purposes. When establishing new routines, some students explicitly allowed time for study breaks, exercise, and meditation before or after study, and often changing scenery by studying at a different place of the house or going for a walk. Dogs played an important role for some students as they encouraged getting out of the house periodically — S53 had four dogs and walked them one at a time. S7 was very explicit in structuring the day to avoid burning out and wasting time, while being kind to themselves: “I suppose, be productive where you can. Then also not beat yourself up for not being productive”. These strategies directly focused on managing affect, motivation in their study, and maintaining physical health. For some students, this involved buying/using equipment like an exercise bike (S49), treadmill (S42), or a rowing machine (S53), using mobile apps for meditation (S29), or adapting their diet (S12). Working out how to take care of themselves took time to figure out, as exemplified by S12, who initially resorted to increasing their alcohol intake before switching to healthier diet and exercise habits. Another student noted:

The drive to work, I've now replaced with doing my physio exercises that prior to COVID, I actually was really struggling to fit in and wasn't doing them consistently and on my drive home, I now go for a walk. I still needed to replace those aspects of what I was doing before COVID just to give my day some structure and some anchor points as well. (S41)

#### Seeking help

Over time, some students’ self-care and wellbeing also resulted in seeking help, but mostly within their existing networks. After missing some exams and failing a couple of units, S7 “went and got help” and recognised the importance of downtime, while S37 said they called a helpline “but that was really no help”, arguing that counselling would bring up a lot of negative emotions. S29 explained that during stressful times, they reached out to friends they met before the lockdown.

To reduce their sense of isolation, some students also made efforts to maintain their social lives through phone or video calls with classmates and friends from outside the university, and S19 reported joining a debate group, which has helped them to become more social: “that loneliness thing made me have to force myself out and it made me realise that I could be more confident in aspects and if anything it encouraged me to join a club activity”.

Some students also reached out for help when they faced study challenges or simply had doubts about content and assessment tasks. This involved actively asking questions on discussion forums or sending emails to their lecturers, often after consulting their peers, attending peer-assisted study sessions organised by the university or asking for help in their own study groups. Others reached out by requesting assignment extensions when they needed, which seemed to be directly related to the ERT conditions. S49, for example, developed migraines from excessive screen time, which led to requesting an extension for taking “a few days off”, whereas S36’s extensions were due to their reduced confidence and motivation during ERT. For S37, requesting an extension was a new and challenging experience, although they reached a positive outcome:

I've never asked for extensions before, but I think I did, a couple of times even, during my last semester and previous semesters. I asked for an extension. Some that I never needed, but it was nice to know I had time to do it. I still submitted on time though. (S37)

Finally, some students also reached out when facing technical issues, particularly during remote exams. For instance, S47 had problems with scanning exam responses when their printer stopped working, but they were able to contact IT support who recommended “to take photos as evidence of completing the exam” and guided them through the process for a supplementary exam.

### Navigating life-integrated learning: the interplay between the dimensions

We coined the term *life-integrated learning* to describe the coming together of living and learning virtually from home. This term reminds us that learning is always anchored in specific practice arrangements and social and material conditions. We cannot fully understand our students’ engagement in their studies without a sense of these temporal dimensions of agency, as illustrated by their situated and bounded actions. Our data show new projections of being an online learner alongside complex negotiations and attempts to re-establish spatial and material cues for studying and overcoming distractions in the absence of dedicated spaces for study such as libraries. While there might be “patterns” of actions, these remain fundamentally situated, specific to student circumstances and lives.

Early in any change, when iterative agency (routines) is no longer suitable, students are oriented to the new arrangements through complex adaptations, negotiations, and actions in the present, which can be emotionally charged as illustrated by S29:

It was very stressful at the start because obviously everyone was transitioning, teachers, lecturers, everyone, and assessments were due quite soon. We really had to like, just completely change where we were getting the information and how, and when it was due. Yes, it was just very full-on at the start but once we understood that these are the places you have to go to find the information. This is when you submit it, this is how to get help for it. Then it was a bit easier to process but before that was just very, very stressful. (S29).

However, these practical-evaluative acts can become part of iterative agency over time, and as the emotional burden dropped, students expressed hope through imagining a better future supported by current practical-evaluative acts and routines. For example, S36 noted that:

It's been many, many weeks since we transitioned to online learning so I think I'm at a stage now where I'm comfortable with online learning as well as entering a new trimester. Entering the second trimester, I actually knew I was being online. I'm going to do online learning right from the word go. So, I was able to set my routines around that and my expectations around that as opposed to last trimester where we were on campus, and then we transitioned into online. I think now I've got a better expectation, and I think I'm coping a lot better with the online learning.

These students illustrate the interlocking nature of the dimensions of agency. As circumstances changed and students needed to adapt, they drew from old routines, to the here-and-now, to translate into a short-term projected future. It also underlines the entangled nature of the various aspects of practical-evaluative agency: the student is having to simultaneously reorganise their study routines, manage stress, and learn where to find help.

## Discussion

Our findings show the multiple actions and dimensions associated with student agency during ERT. Given the uncertainty of ERT, students described limited projective agency. They took a narrow view: invoking strong vocational hopes of the future to overcome current motivational slumps and to act in the present. Iterative agency was also limited due to changed circumstances; however, we saw some routines from the past carry over for students who had studied and/or worked online previously. This was particularly true for those who had studied online, and for students who were familiar with online meeting technology from work settings. Both illustrate the challenges faced by students who may not have familiarity with projected futures following graduation or university routines to support their agency. Students’ practical-evaluative responses were complex adaptations that required figuring out alternatives of action, often resulting in new forms of iterative agency by establishing new routines. They illustrate the way that students can actively and inventively respond to challenges and seek to adapt (and negotiate) their study to the broader challenges of their life circumstances.

For the majority of students, routines of campus and study were significantly disrupted. This necessitated new ways of structuring self, time, space, and relationships. The uncertainty and strongly negative emotional impact associated with the pandemic during 2020 may have driven students’ capacity to keep focussed on the present. This may be particularly true in Melbourne where lockdowns were long by international standards (Wahlquist, 2021) and could in part explain why most of our students’ actions for engaging with ERT mostly occurred within the practical-evaluative dimension. Moreover, these adaptive responses are interwoven with affective responses as students needed to deal with a lack of motivation and a sense of isolation and anxiety at the time that they were required to remain productive in their studies, work, and family lives.

Of course, students always actively interact with, and curate, spaces for learning. However, on campus, there are clear social and material affordances signalling study — such as quiet library zones, books, lecturers, and lecture theatres. With ERT, there was a narrowing of possibility, so that study became circumscribed by the home, the device, and the student. As Alarcón López et al. (2021) found: “The movement from the university (public space) to the students’ homes (private space) made visible different and sometimes conflicting logics: the private family logic” (p.419). This accords with Massey’s (2005) theorisation of space shaping social relations and practices. The spatial cues through the built environment, proximity of peers and teachers in university spaces, introduce a form of accountability (Wilson et al., 2020) that is not readily available when studying from home. Students enforced their own forms of accountability and ordering through material means including pinning timetables and to-do lists on their walls or keeping them near their computers.

In our research, those with the means to commandeer spare rooms, purchase new equipment, etc. were better placed to engage with ERT. Those with shared spaces, without the structural support of extra space or money, required more delicate and in-the-moment negotiations to successfully undertake study activities. Difficult living situations were most likely to reduce confidence in learning during ERT (Bartolic et al., 2022; Guppy et al., 2022). However, students seemed to build new routines for online learning as a result of their early experiences that perhaps, in part, explain the recovery in self-efficacy and wellbeing reported by other researchers as the pandemic continued (Camfield et al., 2021; McKay et al., 2021).

Our study emphasises the importance of spatial and social cues to students’ capacity to organise their daily routines. There was also additional emotional work – needing significant negotiation and workarounds. This aligns with reports of staff experiences, who had to negotiate the use of space with others in their household which wouldn’t typically be part of their work (Littlejohn, 2022). This may be one reason why ERT was harder for the participants in our study who had not taken up remote/online study before. It may be that students who previously chose to study remotely already had grappled with these issues (Crawford & Emery, 2021; Randi & Corno, 2021; Stone & O'Shea, 2019), but these students’ experiences have only recently become of interest in the ongoing shift to supporting the success of non-traditional students.

### Implications

We came to this study to “think with” COVID-19. This study outlines how students are highly adaptable; their ability to agentically adjust their circumstances in the face of disruption is admirable. It also reveals the challenges facing those students whose study remains heavily constrained by time and space. It not only illustrates how hard students work to create study routines in the present but also illustrates how for some, in times of challenge, iterative and projective forms of agency may be limited. It is interesting to speculate that when presented with emotionally difficult times with limited time and space — for example, serious illness or trauma or particularly burdensome caring responsibilities — students may focus on the “here-and-now”. In these instances, learning at home may present the same wellbeing challenges, experienced in lockdown. Moreover, when some of our students find themselves living in lockdown-like situations, such as isolation or remote study, our study suggests that this will be exacerbated by a lack of resources, space, or prior higher education experience.

We suggest institutions should more proactively support students who face challenges with life-integrated learning. Our students found their own adaptations and others may find them useful. In particular, the value of a study routine, building iterative agency, appears to be important. Checking in with those who are studying online for the first time to ensure that they have time and space at home for study may be worthwhile. While self-care may be dismissed by some students as a kind of amorphous and unrelated activity, this study illustrates that balancing emotions through non-study activities can have a significant impact. However, individual self-care can only go so far in the face of significant systemic challenges, arguably, like using a bandaid to stem an arterial bleed. But in collaboration with systemic supports, expanded student networks, and reduced barriers to help-seeking, such self-care efforts may prove to be valuable.

In recent years, the discourse has moved away from a holistic rites-of-passage student education, to a flexible, techno-savvy, and anywhere-and-anytime imaginary (Bearman et al., 2022). Our study clearly shows that much ordering and negotiation was necessary to engage in ERT in a situated rather than anywhere/anytime manner. It may be time to re-emphasise a kind of holism here, to return to the mantra that university is about the whole self, not just acquiring a degree irrespective of modality. Self-care may be more palatable when it is seen as a necessary and holistic part of education, rather than an add-on. Indeed, this study suggests that if we are moving to life-integrated learning, we must view learning and life as being two sides of the same coin.

### Strengths and limitations

Data collection took place during particularly restrictive lockdowns so student accounts are contemporaneous, not retrospective. It was conducted at the beginning of the second trimester of ERT roughly 4 months into lockdown. Data collection was limited to interviews, but we focussed on activities — what they actually do studying during ERT. We note that students volunteering to take part in our research is a particularly agentic act. Students who struggled with seeing or taking up opportunities or were so constrained with time, mental health, or other aspects of life, study, and work may not have been able to take part in the research. Although some students did describe short periods of loss of agency (“I just cried, I told you. There’s nothing much I can do” (S37)), the nature of the study and questions elicited their agentic acts in and around these periods. Certainly, many of our students showed they had agentic opportunities (such as the means to buy equipment or arrange space), despite the added layer of the constraint of the pandemic. Wanting to anchor our approach in stories of rich experience to understand the environment may have prompted more of the here-and-now type data rather than future projections. This study was undertaken at a single university, which already had a strong online and flexible learning presence (unlike other institutions locally and globally), so the differences may have been less obvious in this cohort and may limit transferability to other contexts. Future research may seek to identify what agentic activity is retained post-pandemic as hybrid learning takes hold. In addition, research may investigate how forms of projective agency, quite limited in our study, change in post-pandemic times across diverse populations.

## Conclusion

The pandemic ERT response changed the social, spatial, and temporal arrangements of higher education. Our findings speak to students’ adaptability and agency. The majority of student actions were oriented to the here-and-now, that is, the practical-evaluative dimension of agency through the following: (1) organising self, space, time, and relationships; (2) self-care; and (3) seeking help. While this might be because of the initial COVID-19 lockdowns, when people tended to live “in the moment” and future projections and past experiences were rendered less salient, it may also be reflective of living in emotionally, socially, and materially difficult times. These acts though can become parts of rituals and future repertoires of life-integrated learning.
